# Management of Cartilage Conduction Hearing Aids in Pediatric Patients

**DOI:** 10.3390/audiolres13060076

**Published:** 2023-11-06

**Authors:** Satomi Yakawa, Tomoko Sugiuchi, Rika Myojin, Kiyoko Sato, Takako Murakami, Yuki Miyoshi, Yuichiro Sugio

**Affiliations:** 1 Department of Otolaryngology, Sugiuchi Clinic, 2-7-4 Jiyugaoka, Meguro-ku, Tokyo 152-0035, Japanrika199865@outlook.jp (R.M.); 2 Kotoba no Mori—Child Development Support and After-School Daycare Facility, 2-7-4-2F Jiyugaoka, Meguro-ku, Tokyo 152-0035, Japan; 3 Department of Otolaryngology, Kanto Rosai Hospital, 1-1 Kizukisumiyoshi-cho, Nakahara-ku, Kawasaki-shi 211-8510, Kanagawa, Japan; 4 Department of Communication Sciences and Disorders, Faculty of Health and Welfare, Prefectural University of Hiroshima, 1-1 Gakuen-cho, Mihara-shi 723-0053, Hiroshima, Japan; 5 Kawasaki City Central Rehabilitation Centre, 3-16-1 Ida, Nakahara-ku, Kawasaki-shi 211-0035, Kanagawa, Japan

**Keywords:** atresia, cartilage conduction hearing aids, conductive hearing loss, infant

## Abstract

Forty-nine children who started wearing cartilage conduction hearing aids (CC-HAs) before completing elementary school (17 with bilateral hearing loss and 32 with unilateral hearing loss) were followed-up and examined. The wearing and utilization status of the CC-HA and its progress to date were evaluated. In addition, 33 participants who purchased the CC-HAs were interviewed to assess the wearing effect. Eleven of seventeen children with bilateral hearing loss and 25 of 32 children with unilateral hearing loss continued to use the CC-HAs. In terms of wearing effect, a good wearing effect was reported, even by those with unilateral hearing loss. In cases where it was difficult to wear CC-HAs stably with pasting or ear tips, it was possible to fix them stably using commercially available hair bands and eyeglass vines. In two cases, the CC-HAs were worn from infancy. With ingenuity and appropriate educational and medical support, it is possible to wear CC-HAs from infancy.

## 1. Introduction

Acoustic energy traveling from a cartilage conduction transducer to the cochlea reportedly occurs via three different pathways [1]. The first is the air conduction (AC) pathway from the transducer to the eardrum, which includes the resonance effect in the canal (air pathway) because the transducer also generates a low-level air-borne signal. Vibrations of the aural cartilage and tissue surrounding the external auditory canal generate sound in the ear canal that reaches the eardrum via the AC. The second pathway, the bone conduction (BC) pathway, involves the transmission of skull bone vibrations induced by a transducer to the cochlea [2]. 

The third pathway, first reported in 2004, involves bone and cartilage conduction via the skull from the transducer to the cochlea (cartilage–bone pathway) [3]. Unlike AC, mechanical signals can be transmitted directly to the tissues during cartilage conduction (CC). The CC also avoids the impedance mismatch between air and skin, gaining transmission advantages in the atretic ear over the AC. 

Air and cartilage–bone pathways are common routes that operate based on the same principles that pertain to regular air and bone conduction hearings, respectively. AC and BC hearing aids (HAs) use the first two types of sound transmission pathways. In contrast, the cartilage–air pathway is not a common sound conduction route. Applying vibrations generated by gently placing a transducer on the auricular cartilage can transmit audible sounds with clarity similar to that of AC or BC, leading to the development of a new type of hearing aid called a cartilage conduction hearing aid (CC-HA) [1,4]. However, the exact acoustic details of CC-HAs remain unclear.

CC-HAs can amplify and transmit sound signals to the inner ear by simply attaching a vibration generator to the skin of the auricular cartilage [5,6]. In contrast, bone conduction hearing aids (BC-HAs) also use a vibration generator placed on the body but require strong pressure and fixation on the temporal bone [7,8,9,10,11]. Both types of HAs are suitable for individuals with conductive or mixed hearing loss who cannot wear AC-HAs and for those with good bone conduction thresholds, such as individuals with microtia or external auditory canal atresia. The greatest advantage of using CC-HAs in clinical settings is that the transducer is significantly smaller and lighter than the conventional BC-HA. Moreover, it does not require compression fixation, which enables the CC-HAs to be attached to the skin to deliver sound vibrations to the ear. Furthermore, using CC-HAs eliminates the necessity of surgery and provides users with cosmetic advantages [12].

Since the release of CC-HAs, the attachment-only method has become the preferred option and has gained popularity as an alternative to BC-HAs [1,5,6,8,13,14,15,16,17]. Nishiyama et al. [17] investigated adult candidates eligible for using CC-HAs and concluded that patients with external auditory canal stenosis or anotia are the most suitable candidates. They also reported positive results in children with similar ear conditions [18]. A recent clinical trial involving CC-HA use among children has revealed that almost all parents of the patients reported satisfaction with the performance of the device and an improved daily communication in children with hearing loss [19].

Since 2020, safety measures such as battery boxes have been fully integrated, allowing the use of the device even for children under three age of 3 years [18]. In the case of infants, there are many opportunities to re-examine the possibility of using CC-HAs after starting BC-HAs; however, there have been no reports of initiating the use in infancy. Therefore, in this study, we investigated the usage and wearing progress of CC-HAs in infants and toddlers, presented cases of their application from infancy, and discussed case-specific suitability of various CC-HAs based on the unique requirements of each child.

## 2. Materials and Methods

### 2.1. Participants

This study enrolled 49 children (17 with bilateral hearing loss and 32 with unilateral hearing loss), including 28 boys and 21 girls, in whom trial hearing was initiated using the CC-HA before primary school age at our hospital. The guardians/parents of these children requested the use of CC-HAs. Trial hearing was initiated between the ages of 0 (3 months) and 11 years in children with bilateral hearing loss and between 0 (6 months) and 10 years in those with unilateral hearing loss. The mean age of the participants was 5.02 ± 2.71 (SD) years.

**Figure 1:**
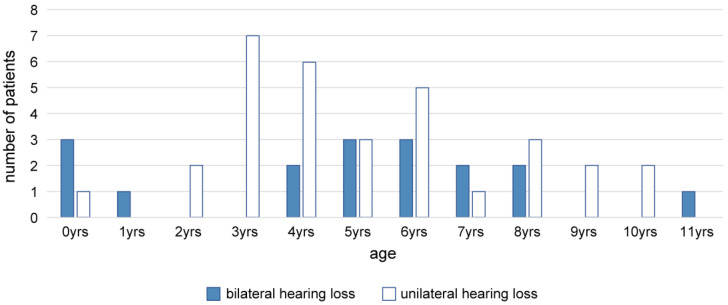
Age distribution of the participants at the time of the initiation of trial hearing (n = 49).

**Figure 2:**
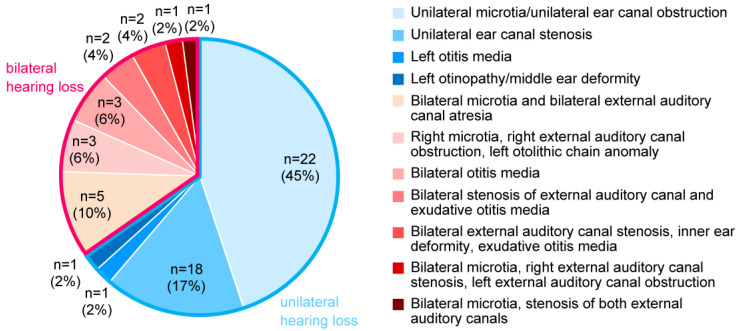
Diagnoses of the fitted ears.

Figure 1 shows the age distribution of the participants at the time of trial initiation. Figure 2 presents the ratios of diagnoses of ears fitted with CC-HAs (HB-J1CC, HB-A2CC, RION Co., LTD., Tokyo, Japan).

Among the participants with bilateral hearing loss, eight had conductive hearing loss, eight had mixed hearing loss, and one participant could not undergo bone conductometry. Among the participants with unilateral hearing loss, 29 had conductive hearing loss, one had mixed hearing loss, and two could not undergo bone conductometry. Among the participants with bilateral hearing loss, two had chromosome 21 trisomy, and the remaining participants each had Treacher Collins syndrome, chromosome 18 trisomy, FOXP1 syndrome, and Primrose syndrome.

The presence of a history of HA use was not observed in cases of unilateral hearing loss, and it was only observed in five of the 17 cases of bilateral hearing loss (BC-HA: two cases at the ages of 6 and 7 years, unilateral-AC-HA: one case at the age of 5 years).

### 2.2. Hearing Assessment

Auditory thresholds were assessed by an experienced audiologist in a standard soundproof room using a commercially available audiometer (Model AA-HI, RION Co., LTD., Tokyo, Japan). Pure-tone thresholds were determined using over-ear headphones (125 Hz to 8 kHz) to assess the air conductance thresholds and a calibrated bone-conducting transducer (500 Hz to 4 kHz) to assess the bone conductance thresholds. The sound field (SF) thresholds were evaluated to assess the effects of the CC-HAs. Complementary and non-complementary hearing thresholds were assessed by introducing an azimuthal angle of 0° and transmitting warble tones from a loudspeaker positioned 1 m away from the participant. As the CC-HAs were fitted on only one side in participants with unilateral hearing loss, noise masking was provided to the other ear through headphones such that the test tone could not be heard. The complementary hearing threshold for CC-HAs could not be accurately assessed in participants with unilateral hearing loss; therefore, the hearing threshold was used as the reference value. Behavioral hearing tests, such as behavioral observation audiometry (BOA) and visual reinforcement audiometry (VRA) were used to assess the hearing ability if the participant was too young to undergo the hearing tests described above. Behavioral hearing tests were performed in a manner similar to those used in previous reports from Japan [20].

### 2.3. Adjustment and Fitting of the Devices and Ethical Standards

The devices were fitted at the Sugiuchi Clinic. Participants or guardians were provided explanations regarding the CC-HAs. Concurrently, ENT examinations, hearing tests, and imaging were conducted to confirm HA history and indications prior to initiating the trial hearing. Trial hearing with the fitted CC-HAs was continued for 1–3 months free of charge, and the participants were instructed to assess the usefulness and comfort of using the CC-HA in their daily lives during the trial hearing period. We provided the participants with the option to extend their trial period until a satisfactory agreement was reached, which could be approximately 6 months.

The initial adjustment of the HAs was performed using the sedation level version 5 (DSL v5) procedure [21]. This procedure and the determination of the hearing threshold for the CC-HAs were similar to those for the AC-HAs. After the hearing aid was tested in an outpatient setting, the hearing threshold was assessed, and the gain and output of the HAs were predicted; fine adjustments were made if necessary. Subsequently, the trial hearing was continued for 1–2 weeks in a real-life setting. The fitting conditions and effectiveness of the HAs were evaluated during this period, and the HA was readjusted based on the user’s wishes. The listening tests and adjustments were repeated until the participant or guardian decided whether to purchase the HAs without any psychological burden on the participant.

**Figure 3:**
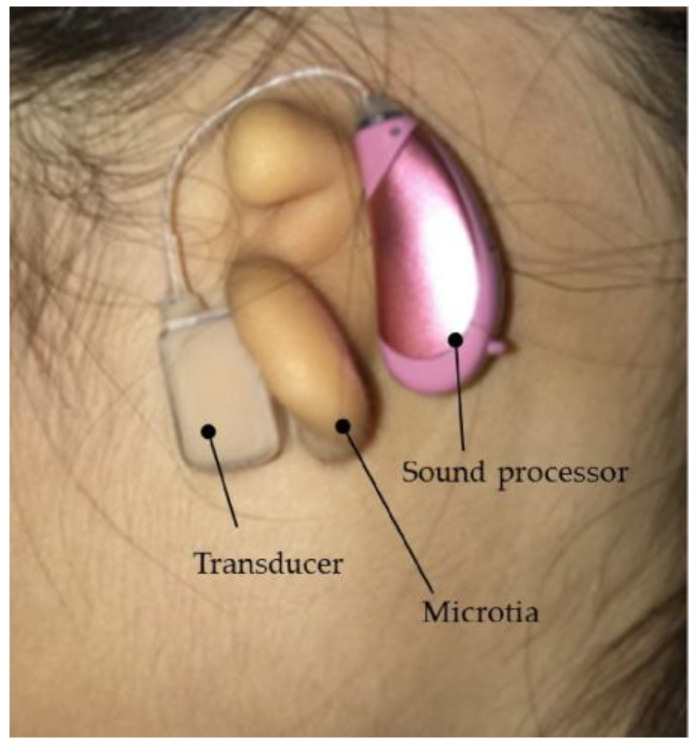
Profile view (left side) of a patient with congenital external ear canal atresia fitted with a cartilage conduction hearing aid (CC-HA). The transducer and sound processor components of the CC-HA (HB-J1CC, RION Co., LTD.; Tokyo, Japan) are attached to the skin using a double-sided adhesive tape.

**Figure 4:**
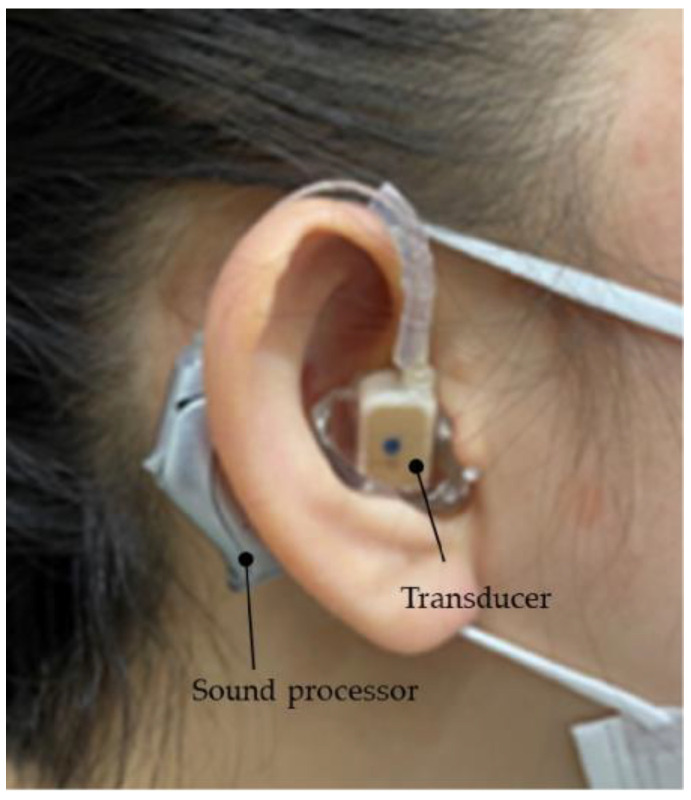
Profile view (right side) of a patient with congenital external ear canal atresia fitted with a cartilage conduction hearing aid (CC-HA). The transducer (with ear tips) is attached to the skin using a double-sided adhesive tape (sound processor components of the CC-HA: HB-J1CC, RION Co., LTD.; Tokyo, Japan).

The vibration terminal (transducer) of the CC-HA was attached to the skin overlying the tragus cartilage and fixed with a double-sided adhesive tape. As the morphology and location of the tragus and auricular cartilage were not well mapped in patients with microtia or congenital aural atresia, the transducer was carefully applied to the skin overlying the cartilage near the assumed location of the tragus, with a subtle concavity (Figure 3). The sound processor of the CC-HA was affixed to the skin overlying the posterior auricle using double-sided adhesive tape. An earmold (referred to as an ear tip) was fabricated if the attachment with the adhesive tape was difficult or if the attachment was unstable, and the transducer was attached to a depressed area such as the cavity of the concha (Figure 4).

In principle, the hearing test was initiated as described above. CC-HAs were attached to the posterior parts of both the auricles in participants with bilateral hearing loss. The CC-HAs were fitted to the affected ear in participants with unilateral hearing loss, similar to those with unilateral congenital auricular atresia. The two CC-HAs were fitted for participants with bilateral hearing loss, similar to those with bilateral congenital auricular atresia. The hearing test conditions were the same for participants with one and two CC-HAs.

Explanations regarding the indicated HAs, such as bone-conduction HAs, BAHAs (Cochlear Limited, Sydney, Australia), and the ADHEAR system (MED-EL, Innsbruck, Austria), were provided, and demonstrations via test hearing were also provided, if possible, upon request. Furthermore, the staff at the rehabilitation institution provided information regarding the need for HAs, and the model and adjustment of HAs. The final decision regarding the purchase of HAs was made by the parents based on the HA use thresholds and the combined observations and evaluations of the parents and caregivers.

This study was conducted in accordance with the “Ethical Principles for Medical Research Involving Human Subjects” [22] as stated in the Declaration of Helsinki and approved by the Ethics Committee of Kanto Rosai Hospital (Approval No.: 2023-1). The details of the study were posted in the clinic examination room. Verbal informed consent was obtained from all the participants and their guardians. The requirement for written consent was waived in accordance with the ethical guidelines for medical and health science research involving human participants [23]. Information regarding the study, including its purpose and use, was made publicly available or notified to the research participants. Participants and their guardians were informed that they could refuse to participate at any time and requested that their data be deleted after the start of the study. This information was included in the medical records of each participant.

### 2.4. Purchase Rate and the Evaluation of Cases That Did and Did Not Purchase CC-HA(s)

The overall purchase rate was evaluated and the participants were divided into two groups based on whether the CC-HA was purchased: purchase and non-purchase groups. Information regarding age, sex, condition of the ear fitted with the HA (affected or good ear), and mean hearing thresholds (500, 1000, and 2000 Hz) of the participants were collected and used for comparison. Participants who were too young to undergo hearing assessments such as sound field thresholds were excluded from the study.

### 2.5. A Simple Way to Improve Hearing Aid Fixation

The following methods were used when the HAs could not be stabilized by attaching CC-HA transducers and sound processors.

**Figure 5:**
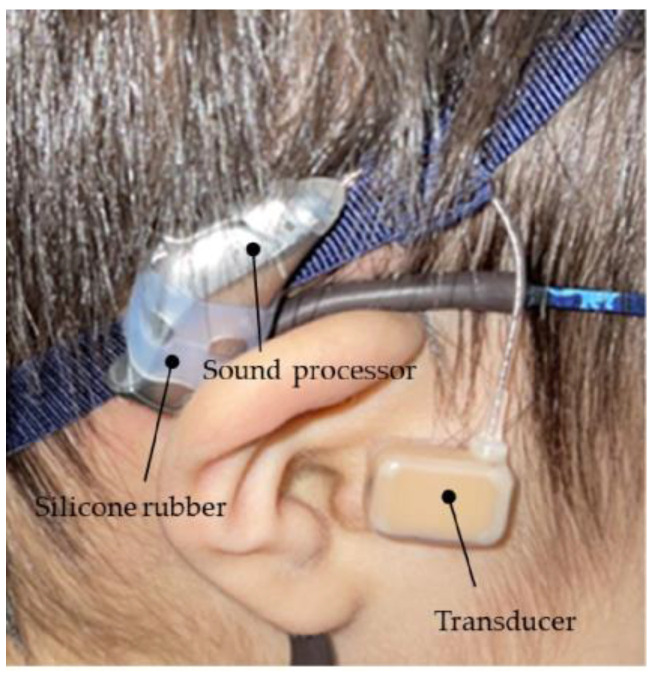
Profile view (right side) of a patient with Downs syndrome who has bilateral external ear canal stenosis with exudative otitis media and congenital external ear canal atresia fitted with a cartilage conduction hearing aid (fixed onto the headband with silicone rubber). The transducer is attached to the skin using a double-sided adhesive tape.

**Figure 6:**
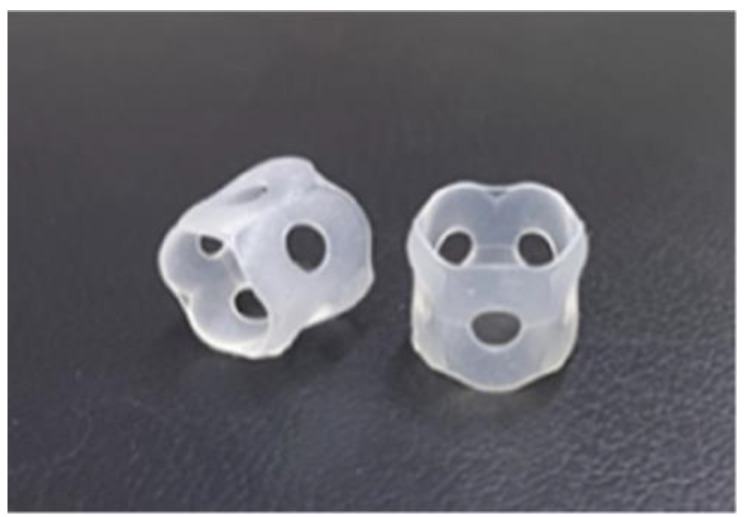
Silicone rubber, which is sold as a stationery item, can be used for attaching the main body of the hearing aid to the temple of the glasses or headbands.

Use of hairband: In this method, a silicone rubber was sewn onto a commercially available flat rubber-like hairband to which the hearing aid body was fixed. The transducer was subsequently attached to double-sided adhesive tape (Figure 5 and Figure 6).

**Figure 7:**
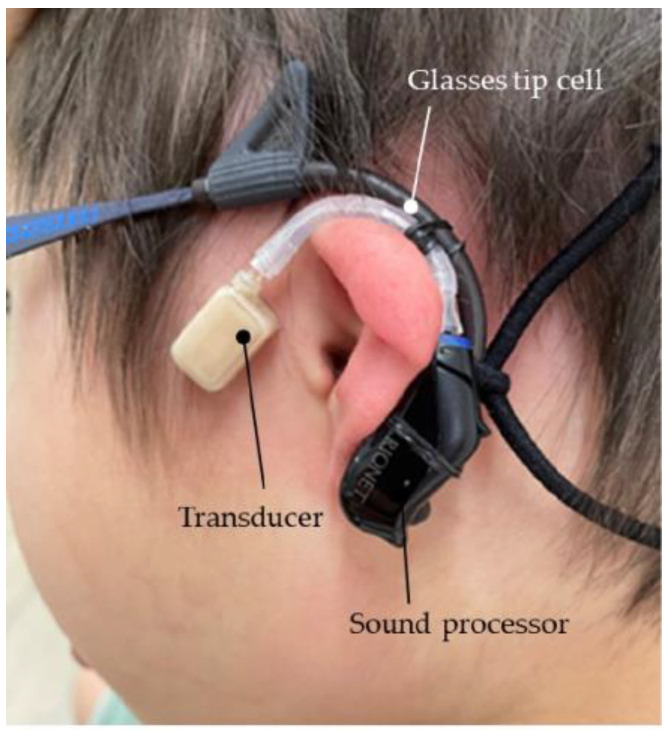
Profile view (left side) of a patient with bilateral external auditory canal stenosis with exudative otitis media wearing a cartilage conduction hearing aid fixed to the temples of glasses. The transducer is attached to the skin using a double-sided adhesive tape.

Use of eyeglasses: In this method, the sound processor of the CC-HA was fixed to the temple of the glass using rubber or silicone rubber, and the transducer was subsequently attached (Figure 7).

### 2.6. Evaluation after Purchase

The participants or their guardians who purchased the CC-HAs were interviewed during the consultation to understand their post-purchase status, and the participants were evaluated. The questions included the duration and effectiveness of HA use and requests for HA use. Questions and options regarding the duration and effectiveness of HA use were determined in advance.

## 3. Results

### 3.1. Purchase Rates and Differences between the Participants Who Did and Did Not Purchase CC-HAs

Among the 17 participants with bilateral hearing loss, 11 (64.7%) had purchased a CC-HA. Among the 32 patients with unilateral hearing loss, 25 (78.1%) had purchased CC-HAs. We examined the average hearing thresholds according to age, bilateral hearing loss, and unilateral hearing loss and divided the participants into purchase and non-purchase groups; however, no significant difference was observed between the purchasing and non-purchasing groups in terms of any of the measured characteristics (Table 1). Among the participants with bilateral hearing loss who purchased CC-HAs, three (17.6%) used the CC-HA only on one side, whereas AC-HAs were used on the opposite side with external auditory canal stenosis or inner ear/middle ear anomalies. None of the participants reported experiencing any complications with the use of CC-HAs, such as skin irritation. Representative cases of varying hearing conditions and fitting requirements are presented as case reports.

### 3.2. Participants Who Did Not Purchase CC-HAs

There were various reasons for not purchasing the CC-HAs. Among the five participants with bilateral hearing loss in the non-purchase group, the parents of a 3-month-old infant selected the BC-HA because of its ease of attachment and detachment. The parents of the 6-month-old infant started using the BC-HA at a different hospital. The parents of a 7-year-old patient with bilateral microtia who was currently using a BC-HA (with a fabric headband) wished to continue using the BC-HA until ear reconstruction surgery. A child with right external auditory canal stenosis and a left middle ear anomaly was found to have an enlarged right external auditory canal during the process of making an ear impression for CC-HA ear tip fabrication; thus, AC-HA was selected.

Furthermore, a 4-year-old child with chronic otitis media and immunodeficiency with selective IgG2 deficiency (Primrose syndrome [24]) had the intention to avoid middle ear infections through HA use. However, surgical therapy was successful in achieving a stable usage.

Seven participants with unilateral hearing loss in the non-user group refrained from purchasing HAs because of their personal reluctance to use it.

### 3.3. Aided and Unaided Hearing Thresholds of the Purchase and Non-Purchase Groups

**Figure 8:**
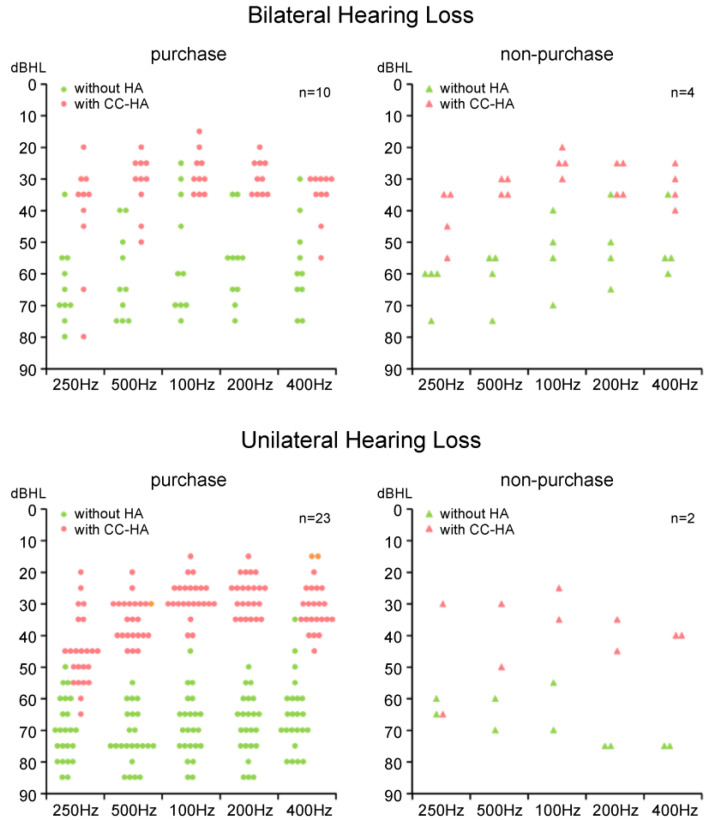
Average unaided and aided sound field hearing thresholds in bilateral and unilateral hearing loss of participants in purchase and non-purchase groups. The dots on this graph represent the raw data for each frequency for each case.

Some participants were too young and it was very difficult to measure the hearing thresholds, even at the sound field threshold, and a few participants, especially in the non-purchase group, chose not to wear the HAs early and did not undergo measurement. Figure 8 shows the SF thresholds (mean values) without HAs and with CC-HAs at each frequency for the purchase and non-purchase groups, separately for bilateral and unilateral hearing loss. Considering these averages, it can be seen that the SF hearing thresholds were improved by the CC-HAs at all frequencies within each group, indicating auditory effectiveness. There was no significant difference in the SF hearing thresholds between the two groups, except for the unaided thresholds of 2 KHz and 4 KHz in unilateral hearing loss.

**Figure 9:**
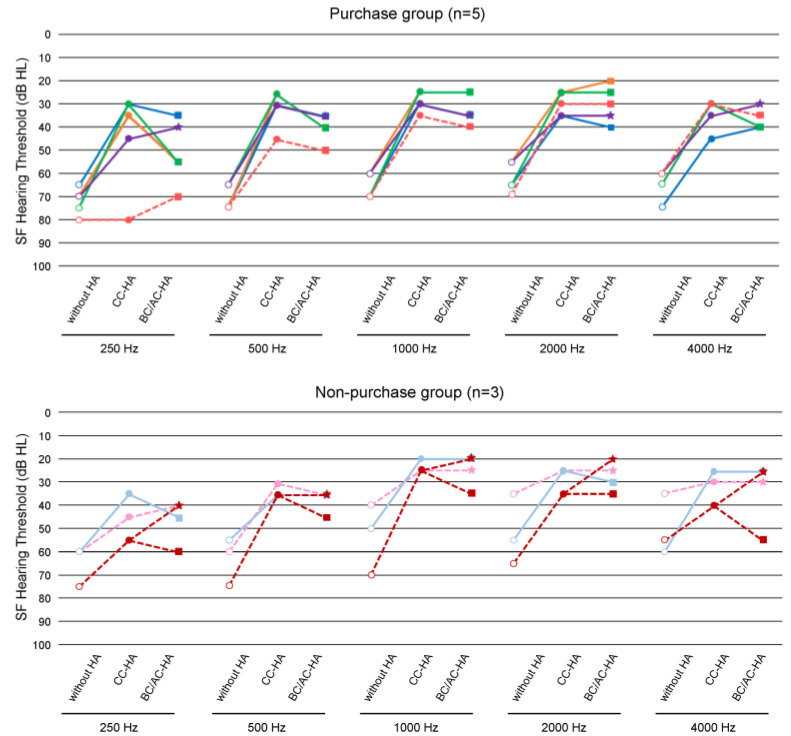
Comparison of SF hearing thresholds in three conditions: without HA, with CC-HA, and with BC-HA/AC-HA. Three-pairs of dots at a given test frequency are data from one participant in the binaural hearing loss group. In both graphs, the hearing thresholds for two or three types of hearing aids are represented by dots of different colors for each case. The differences are shown by connecting the dots with lines of the same color. Open circles indicate thresholds without HA, filled circles indicate thresholds with CC-HA, filled squares indicate thresholds with BC-HA, and filled stars indicate thresholds with AC-HA. The solid line connecting the dots also indicates that the patient was binaural and the dashed line indicates that the patient was uninaural.

Hearing aids other than the CC-HA, that is, the BC-HA or AC-HA, could only be auditioned in the binaural hearing loss group. Of the cases with this comparative hearing loss, the SF thresholds for each HA were measured in eight cases (five with the CC-HAs and three without it). All these patients had better thresholds with the CC-HA than without HAs, with the CC-HA being better than or equal to other HAs in all cases except for one in the non-purchased group (Figure 9). The AC-HA was not available in all cases in the unilateral hearing loss group, and only BC-HA was indicated; however, none of the cases were auditioned due to reluctance to use a catheter-type headset or crimp the bone-conducting terminal.

### 3.4. Post-Purchase Evaluation-Wearing Status According to the Questionnaire Survey

**Figure 10:**
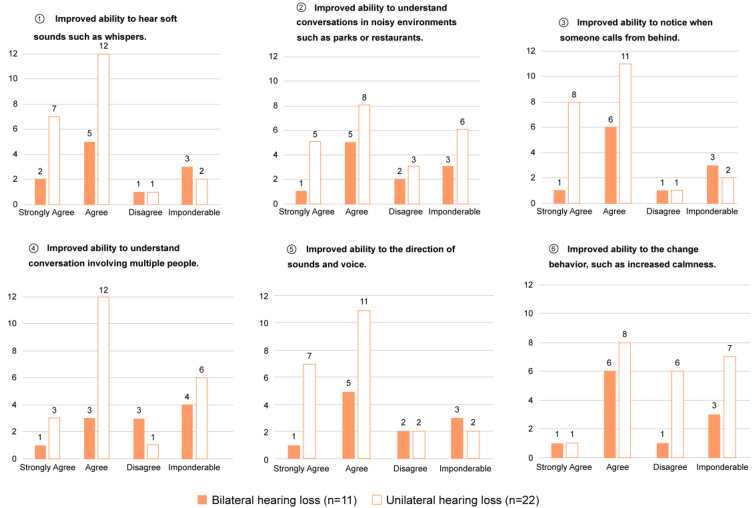
Response to a questionnaire survey to assess the post-purchase evaluation of effectiveness of wearing the hearing aid.

A questionnaire survey was conducted during the post-purchase evaluation of 36 cases, and responses were obtained from 33 participants. All participants with bilateral hearing loss (100%) and 22 of 25 participants with unilateral hearing loss (88.0%) responded to the survey (Figure 10). Three participants could not be interviewed due to discontinuation of follow-up. All participants had unilateral microtia and external auditory canal atresia.

Regarding the effectiveness of wearing, among the statements “noticed voices from behind more easily”, “easier understanding of conversations in noisy places, such as parks or restaurants”, “better understanding of the direction of sounds and voices”, and “easier understanding of conversations with multiple people”, participants with bilateral hearing loss and unilateral hearing loss responded with “strongly agree” or “agree” in over 50% of the cases (Figure 10). However, for “conversations with multiple people”, the agreement rate was low. This tendency was particularly pronounced in patients with unilateral hearing loss.

**Figure 11:**
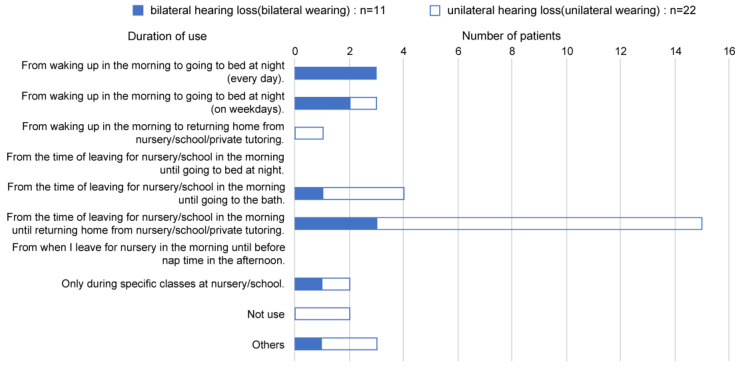
Duration of use of cartilage conduction hearing aids.

The wearing behaviors of the participants are shown in Figure 11. Among the eleven participants with bilateral hearing loss, three participants reported wearing CC-HAs daily from “morning, upon waking up” to “night, before going to bed”, two participants reported wearing the aid in the weekdays “morning, upon waking” to “night, before bedtime”, and three participants reported wearing CC-HAs until “returning home”, and the remaining two participants reported wearing CC-HAs “only at nursery school/school”.

Among the 22 participants with unilateral hearing loss, one participant reported wearing CC-HA from “morning, upon waking up” to “night, before going to bed”, one participant reported wearing CC-HA until “returning home”, and the majority of 15 participants (68.2%) reported wearing CC-HAs from “when going to nursery school/school” to “returning home”. One participant reported wearing CC-HA “only at nursery school/school” and the remaining four participants reported wearing CC-HAs only when spoken to (Figure 11).

**Figure 12:**
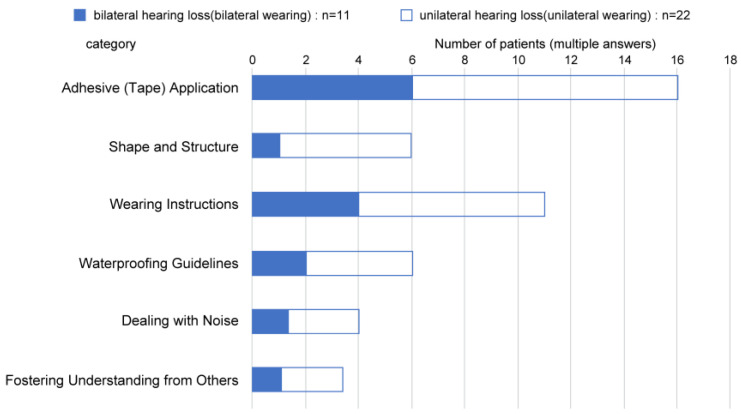
Cartilage conduction hearing aid use (categorized into 6 items).

The requests for HA usage were classified into six categories (n = 46): “concerns regarding adhesives (tape)”, “issues with wearing and handling”, “concerns regarding shape and structure”, “waterproofing concerns”, “concerns regarding background noise”, and “concerns regarding social acceptance”. Among these categories, “concerns regarding adhesives (tape)” were the most common (34.8%), with noticeable responses indicating that the tape tended to become less adhesive over time, owing to sweat. The second most common category was “issues with wearing and handling”, accounting for 23.9% of the responses, with difficulties mentioned in children independently using and putting on the device (Figure 12).

### 3.5. Case Reports

Case 1 was a 3-year-2-month-old girl born at 37 weeks and 1 d of gestation, weighing 2690 g. The patient had multiple malformations (Treacher Collins syndrome), including micrognathia, ptosis, down slanted palpebral fissures, and cleft palate. Tracheotomy was performed after 9 days. The patient required medical care and attended school for the deaf and Kotoba-no-mori.

**Figure 13:**
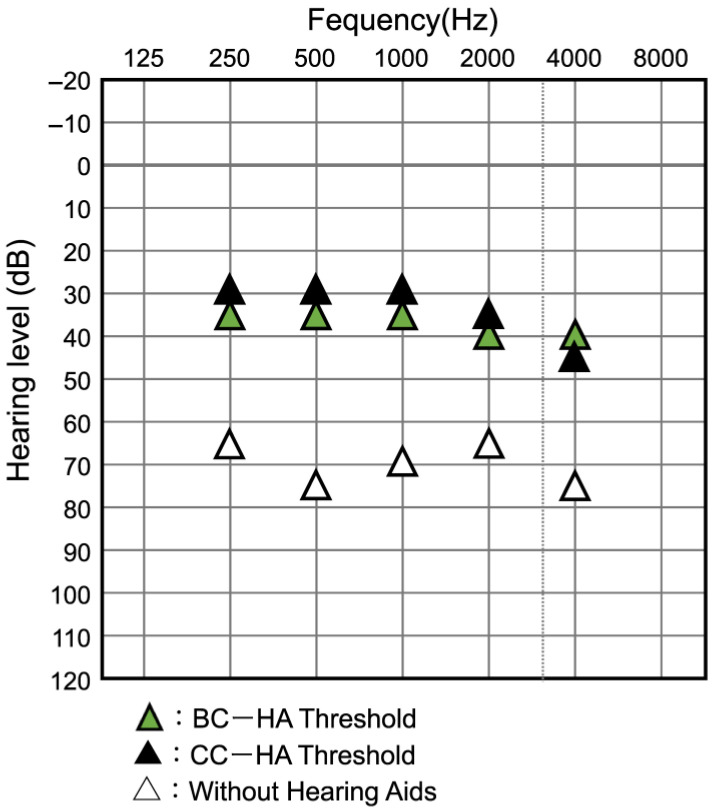
Case 1: Comparison of bone conduction hearing aids’ threshold and cartilage conduction hearing aids’ threshold.

**Figure 14:**
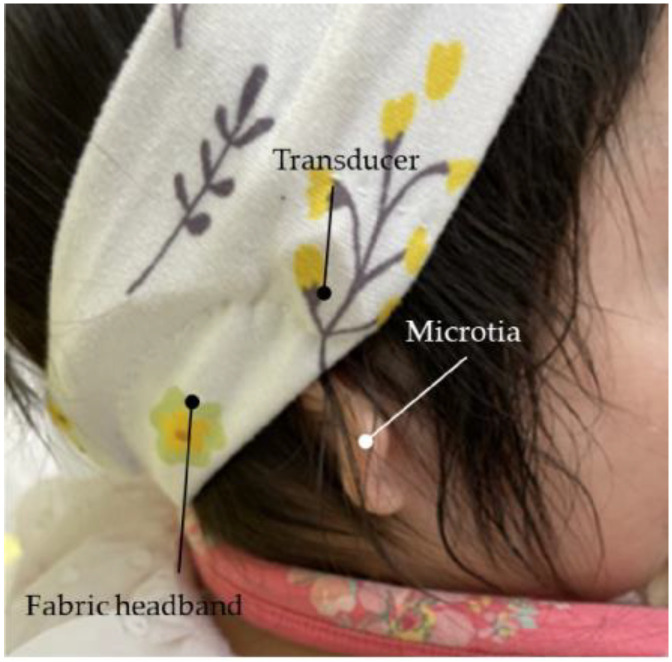
Profile view (right side) of a 6-month-old patient with Treacher Collins syndrome who has bilateral external auditory canal stenosis with exudative otitis media, wearing CC-HA. The main body of the hearing aid and the terminals are sewn into the inside of the headband.

**Figure 15:**
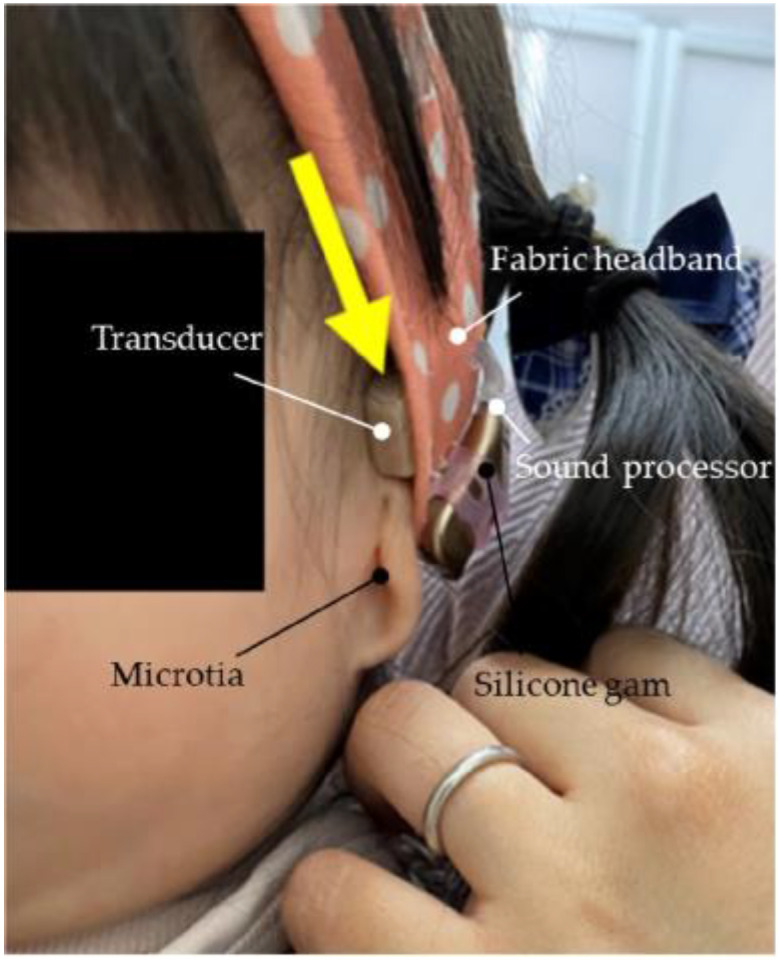
Profile view (left side) of Case 1 (a patient who is over 1 year old with Treacher Collins syndrome with bilateral external auditory canal stenosis and exudative otitis media) using cartilage-conducting hearing aid. The hearing aid is sewn to the headband, the transducer is placed outside the headband, and the transducer is attached with double-sided tape near the ear-pearl cartilage (Yellow arrow indicate the transducer peeking through the headband).

**Figure 16:**
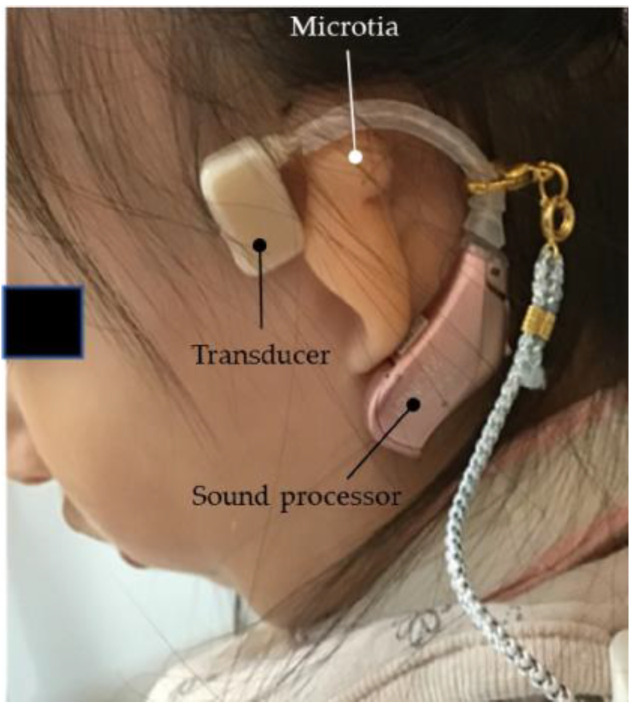
Profile view (left side) of Case 1 (2 years old) with bilateral external auditory canal closure. The terminals and hearing aid body are attached using double-sided tape.

The first visit was at the age of 6 months. Trial hearing with the CC-HA with both the transducer and attached body was initiated, and the clinical course was mostly favorable. Trial hearing with the BC-HA (with a fabric headband) was initiated for comparative listening purposes. Both HAs had similar wearing efficacies (Figure 13). However, the parents of the participant purchased the CC-HA because the participant was able to remove the BC-HA, which could not be stabilized. Additionally, the participants’ mother believed that the CC-HA was easier to work with. Around the age of 9 months, it became somewhat noticeable that the participant could remove the HA immediately after fitting. Therefore, the HA, including the transducer, was sewn into a ready-made hairband (Figure 14) at the age of 14 months. The HA was worn for a longer duration without changes to the threshold and with favorable wearing efficacy. The participant’s mother modified the hairband when the participant was 23 months old. Silicon rubber was sewn to the outer side of the hairband such that the microphone on the CC-HA body was placed outside the hairband. The best wearing efficacy was obtained when a hole was created in the hairband such that the transducer was directly in front of the upper part of the ear, and the hairband was used to cover the transducer (Figure 15). At the age of 26 months, the patient attempted to wear the CC-HA body and transducer via affixation. Initially, the duration of use of the unit was limited. However, the duration increased gradually, and the participant used the device throughout the day (Figure 16).

**Figure 17:**
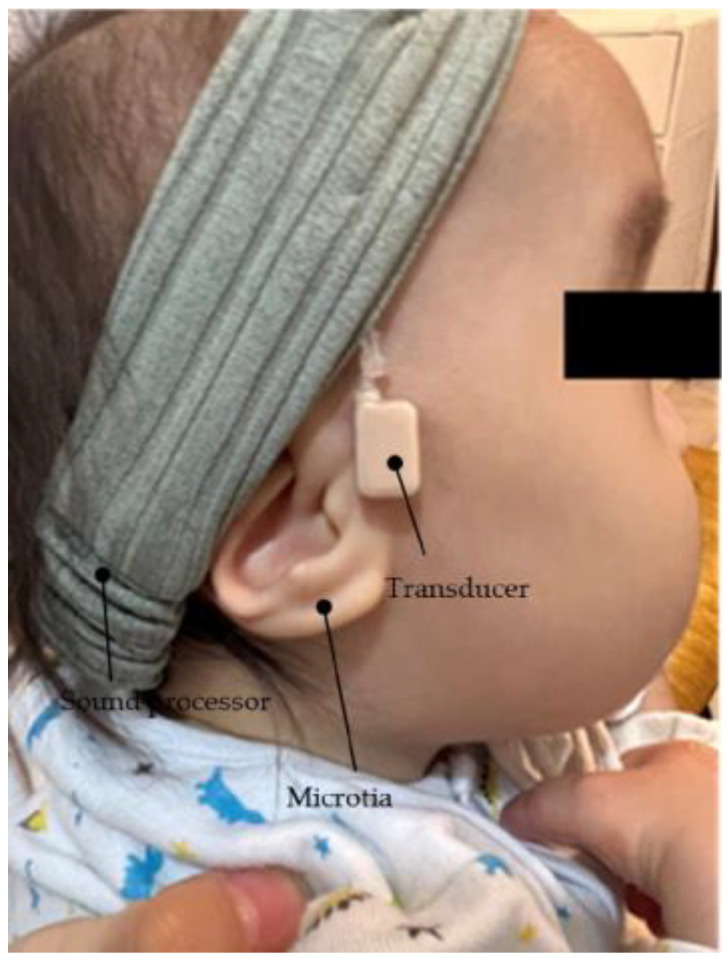
Profile view (right side) of Case 2 (severe bilateral external auditory canal stenosis). The terminals are attached using double-sided tape, and the body of the hearing aid is secured to a fabric headband for wearing.

**Figure 18:**
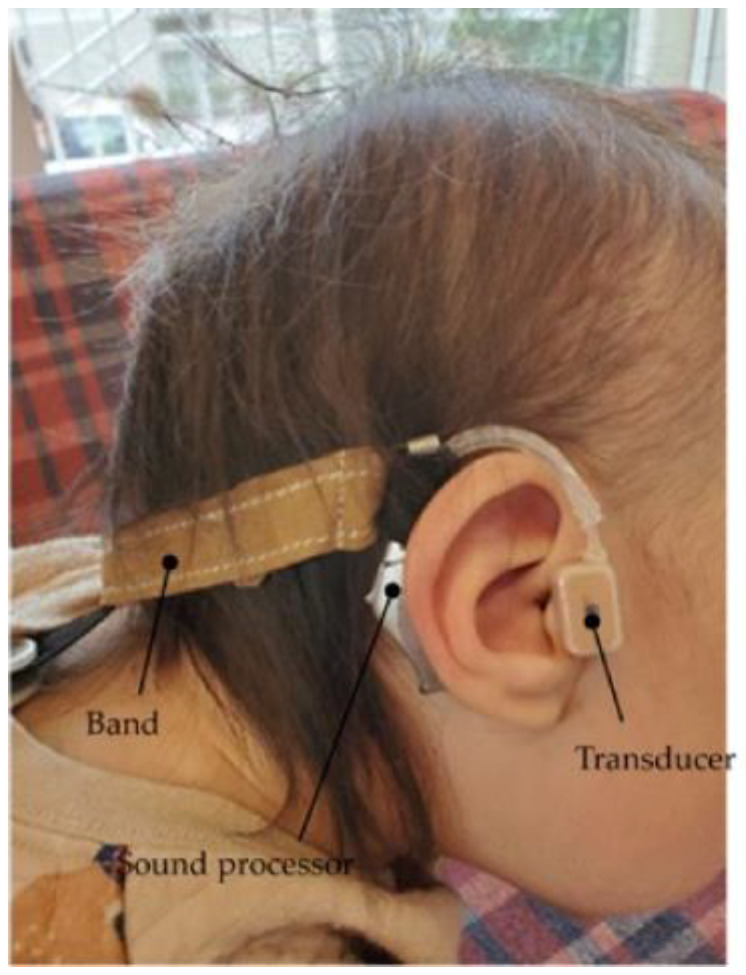
Profile view (right side) of Case 2. The terminals and the hearing aid body are attached using double-sided tape and secured to the back with a mischief-prevention belt.

Case 2 was of a child aged 1 year and 11 months who was born at 38 weeks of gestation and weighed 2398 g. Chromosomal abnormalities (chromosomes 18 and 3) and FOX P1 syndrome were identified. The patient is currently receiving education at a municipal development facility. Significant stenosis was observed in both the external auditory canals during the initial visits at the age of 1 year and 1 month. The possibility of conductive hearing loss was indicated by the auditory steady-state response (ASSR). Following the experience in Case 1, the HA was attached to a commercially available cloth headband, with the transducer placed outside the band for testing purposes (Figure 17). By the age of 1 year and 2 months, the child could wear the HA for approximately 6 h a day, and the mother reported improved sound responsiveness with fewer instances of howling. As the child could remove the headband from the age of approximately 1 year and 5 months, the mother started attaching the main body and transducer directly to the skin for wearing. Currently, for safety reasons, the shoulder area is secured with a short strap, and an HA is used without a headband for a few hours daily (Figure 18). The parents are considering using ear tips in the future.

## 4. Discussion

This study aimed to evaluate the effectiveness and fitting/wearing status of the CC-HA in children with hearing loss, and to examine the indications for HAs in children as they grow older. The primary finding of this study was that it is possible to continuously and stably wear HAs from infancy by devising a fitting method while monitoring developmental status and wearing conditions. The discovery of cartilage conduction pathways has uncovered new possibilities for auditory function.

Continuous monitoring should be accompanied with specific strategies for children with unilateral hearing loss and latent disabilities [25]. Hearing aids are highly effective for treating late-onset conductive hearing loss. Conventional AC-HAs and BC-HAs are effective strategies; however, the former device is not suitable for patients with aural atresia and chronic otitis media, and the latter has esthetic disadvantages because of the requirement of headbands or headsets, causing hesitation in individuals and parents. The small and lightweight CC-HA has minimal resistance and excellent effectiveness, thereby reducing psychological resistance [26]. As a result, CC-HAs are being considered as an alternative to conventional bone-anchored HAs, vibrant sound bridges (VSBs), and cochlear implants, and are frequently used during the pre-surgery stage [12].

We initiated a trial hearing of the CC-HA in 49 cases, ranging from infants to elementary school students, and 36 patients proceeded to decide on and utilize the device. While promoting the suitability of CC-HAs for infants and young children, particularly those with developmental disorders, we encountered difficulties in achieving stable attachment using the recommended methods of adhesion or ear tips alone. Initially, we proposed attaching the transducer using a double-sided tape and securing it further with tape [18]; however, this did not result in a stable attachment. Therefore, taking inspiration from the headbands used for the BC-HA, we collaborated with the participants’ mothers and created prototypes of the CC-HA headbands that quickly made it possible to wear the device. Based on this experience, we found that attaching a hearing aid to the temples of the glasses or using a favorite headband proved to be successful in other cases.

Treacher Collins syndrome (as seen in Case 1), also known as mandibulofacial dysostosis, is an autosomal dominant inherited genetic disorder with an incidence of 1 in 50,000 [27,28]. Common symptoms of this syndrome include hypoplasia of the facial bones, especially the mandible and zygoma, drooping of the cleft palate, lid coloboma, and cleft palate [29]. Conductive hearing loss is observed in 50% of patients and is attributed to malformations of the outer and middle ear [30,31]. Previous studies have reported on auditory rehabilitation in patients using the BC-HAs or BAHAs. The importance of early auditory rehabilitation to ensure the appropriate development of language and learning is well-known [32,33,34,35]; however, the use of BC-HAs is associated with local pain, discomfort, and concerns related to appearance [8,10]. BAHAs require surgery [36,37] and implant protrusion is disadvantageous in terms of appearance [8]. In contrast, the use of CC-HAs is not associated with these problems and is considered an effective alternative to AC-HAs. In this study, a headband was used as an adaptation to the device. Initially, concerns were raised regarding headband shifting; however, no issues were encountered during the study period. Factors such as the child being calm, having minimal body movements during infancy or other life stages, or being at a stage of greater understanding may also have influenced the results.

FOXP1 syndrome (Case 2) is associated with intellectual disability, language impairment, autism spectrum disorder, myotonia, mild dysplasia, and congenital abnormalities of the brain, heart, and urinary system. Hearing loss has also been reported in patients with this syndrome. Lozano et al. [38] reported that all individuals with FOXP1 syndrome must be evaluated for hearing loss and should promptly undergo hearing replacement. CC-HA was effective in the treatment of hearing loss in a patient with trisomy 18. Trisomy of chromosome 18 is the second most frequent autosomal disorder after Down syndrome and 22q11.2 deletion syndrome, with a reported frequency of 1 in 3500–8500 live births. The prognosis is often poor [39,40]; however, marked improvements in vital prognosis have been reported. With the advances in newborn hearing screening tests and early detection of hearing loss, it is desirable for HAs to be worn safely, without burden, and consistently from 0 years of age, even in cases where AC-HAs are difficult to apply, such as in patients with atresia of the external auditory canal.

In this study, five participants with bilateral hearing loss had a history of HA use prior to the CC-HA trial. Four participants used bilateral BC-HAs, and one participant used AC-HA on the side without external auditory canal stenosis. The preference for switching to the CC-HA primarily came from caregivers because of limited wearing time, concerns regarding esthetic aspects, and discomfort caused by the pressure of the BC transducer in the BC-HA. Among the three participants with bilateral microtia and external auditory canal atresia, all participants except one, who was awaiting transition to CC-HA after auricular reconstruction surgery, immediately transitioned to CC-HA. In one participant with unilateral microtia, external auditory canal stenosis, and contralateral ear ossicular malformation, external auditory canal enlargement was observed while making a near impression of the CC-HA, resulting in the selection of the AC-HA. In cases of bilateral hearing loss with microtia, external auditory canal closure, or stenosis since birth, conventional BC-HAs (with cloth headbands) are commonly used by both medical professionals and caregivers because they are easy to wear and readily available. However, CC-HAs offer the potential for stable use from infancy by adapting the wearing method as the child grows and are expected to have wider applications. This adaptation requires repeated prototyping. Moreover, collaboration with parents, especially the mother, is essential, as the mother observes the child’s behavior and experiences the benefits of wearing a HA. The support and involvement of healthcare professionals and caregivers are crucial in increasing the motivation for wearing HAs and encouraging their active utilization.

An evaluation of the post-purchase experience revealed that HAs were used almost throughout the day by participants with bilateral hearing loss. Moreover, both individuals and their surroundings experience the positive effects of their usage. Similar results were observed in the participants with unilateral hearing loss; however, there were some instances of shorter wearing times. Educational and medical support are crucial for the effective use of HAs, particularly in cases of unilateral hearing loss. Additionally, a higher proportion of individuals with unilateral hearing loss reported perceiving the benefits of wearing a HA compared to those with bilateral hearing loss, which may be attributed to the presence of non-usage periods, making the effects of the HA more noticeable. This is believed to reflect the binaural hearing effects reported by Kagaet al. [41]. Regarding the challenges related to HAs, participants with bilateral and unilateral hearing loss identified improvements in the adhesive-wearing method, particularly addressing issues with sweat and difficulties in re-application, as future tasks.

It is assumed that, in the case of bilateral hearing loss, patients are more open to wearing HAs, irrespective of the type, to improve their quality of life and learning abilities. On the other hand, if the child has hearing loss on only one side from birth, caregivers are not keen on providing HAs. In this context, the high rate of device purchase by children with unilateral hearing impairment demonstrated the perceived utility of binaural hearing. The findings also suggested that CC-HAs are one of the most comfortable HAs to wear, and provide reliable and adequately amplified speech.

Participants with unilateral hearing loss had no history of using HAs, and the CC-HA was the first HA selected for these participants because there were limited options available in terms of other models, as they required surgical intervention. In recent years, implants such as the BAHA Attract System (Cochlear Limited), Bonebridge (MED-EL, Innsbruck, Austria), and Sophono (Medtronic, Dublin, Ireland) have been developed. In the case of children, the decision to undergo surgery is primarily made by caregivers (parents). Considering the currently available evidence, identifying one type of HA (AC-HA, BC-HA, or CC-HA) over others is not justified. When deciding on a particular HA, the cases that are more adaptable should be defined. Furthermore, patients’ perceived functional gains, specific hearing status, extent of hearing loss in individual patients, and the cost of HAs should be considered. In pediatric cases, it is also important to consider the developmental status. However, the use of CC-HAs as a policy until the age when the child’s own will can be taken into consideration is also an important option.

## 5. Conclusions

Even in cases where it is challenging to use AC-HAs, such as in patients with external auditory canal closure, the ability to consistently use HAs from infancy is crucial for the development of language and communication skills in children with hearing loss. The CC-HA allows for continuous and stable usage from infancy to early childhood by adjusting the fitting method according to a child’s growth. The use of CC-HAs involves utilizing options such as headbands or attaching the HAs to glass frames. The device is likely to reduce the physical and psychological burden on the infant, as well as on parents or caregivers. Parents of a high percentage of children with unilateral hearing loss in this study purchased and used HAs, indicating positive sentiments toward the device. Collaboration with caregivers is necessary for implementing these adaptations, and an effective use of HAs requires educational and medical support.

## Figures and Tables

**Table 1:** Characteristics of the purchasing and non-purchasing groups.

Characteristics	Purchase Group	Non-Purchase Group	*p* Value
Age at fitting (year, Mean ± SD)	5.3 ± 2.6 (n = 36)	4.4 ± 3.0 (n = 13)	0.376 ^b^
Bilateral hearing loss, Average hearing threshold ^a^ of the better ear (dB HL, Mean ± SD)	46.5 ± 17.3 (n = 8)	56.7 ± 16.7 (n = 3)	0.427 ^b^
Threshold ^a^ of the worse ear (dB HL, Mean ± SD)	65.8 ± 20.3 (n = 8)	61.7 ± 22.5 (n = 3)	0.796 ^b^
Unilateral hearing loss, Average hearing threshold ^a^ of the better ear (dB HL, Mean ± SD)	9.7 ± 5.2 (n = 24)	9.6 ± 3.4 (n = 4)	0.948 ^b^
Threshold ^a^ of the worse ear (dB HL, Mean ± SD)	70.4 ± 12.2 (n = 24)	59.6 ± 16.3 (n = 4)	0.281 ^b^

^a^ Average of AC hearing thresholds at 500, 1000, and 2000 Hz. ^b^ Independent *t*-test.

## Data Availability

Not applicable.
